# Grade 2 Spondylolisthesis at L4-5 Treated by XLIF: Safety and Midterm Results in the “Worst Case Scenario”

**DOI:** 10.1100/2012/356712

**Published:** 2012-10-17

**Authors:** W. B. Rodgers, Jeffrey A. Lehmen, Edward J. Gerber, Jody A. Rodgers

**Affiliations:** Spine Midwest, Inc., Suite 301, 200 St. Mary's Medical Plaza, Jefferson City, MO 65101, USA

## Abstract

Spondylolisthesis is one of the most common indications for spinal surgery. However, no one approach has been proven to be more effective in treating spondylolisthesis. Recent advances in minimally invasive spine technology have allowed for different approaches to be applied to this indication, notably extreme lateral interbody fusion (XLIF). The risk, however, of using XLIF in treating grade II spondylolisthesis is the ventral position of the lumbar plexus, particularly at L4-5. *Objective*. This study reports the safety and midterm clinical and radiographic outcomes of patients with grade II lumbar spondylolisthesis treated with XLIF. *Methods*. 63 patients with grade II spondylolisthesis and spinal stenosis were treated with XLIF and were available for 12-month followup. Of those, 61 (97%) were treated at L4-5. Clinical (VAS, complications, and reoperation rate) and radiographic (anterolisthesis, disk height, and fusion) parameters were assessed. *Study Design*. Data were collected via a prospective registry and analyzed retrospectively. *Results*. Sixty-three patients were available for evaluations at least one year postoperatively. Average pain (visual analog scale) decreased from a score of 8.7 at baseline to 2.2 at 12 months postoperatively. Average anterior slippage was reduced by 73% and was well maintained. Average disk height (4.6 mm pre-op and 9.0 mm post-op) nearly doubled after surgery. Slight settling (average 1.3 mm) occurred over the twelve-month follow-up period. There were no neural injuries and no nonunions noted. *Conclusions*. XLIF is a safe and effective minimally invasive treatment alternative for grade II spondylolisthesis. Real-time neurological monitoring and attention to technique are mandatory.

## 1. Introduction

Spondylolisthesis remains one of the most common indications for surgery on the spine. The efficacy of surgical treatment for this condition has been repeatedly confirmed [1], most notably in the Spine Patient Outcomes Research Trial (SPORT) study [2–4] and as such, fusion is frequently recommended for patients with degenerative spondylolisthesis [5, 6]. While the long-term benefits of surgical treatment over nonoperative care for this indication have been shown, only recently has the cost-effectiveness of this procedure been proven in high-level data [7]. Recent reports have discussed and compared a variety of fusion procedures, including anterior lumbar interbody fusion (ALIF), posterior lumbar interbody fusion (PLIF), [8] transforaminal lumbar interbody fusion (TLIF) [9], and minimally invasive (MIS) TLIF and MIS ALIF [10]. Despite a great deal of investigation, no approach has proven more effective than the others, and no universal treatment guideline can be proposed [5]. 

**Figure 1:**
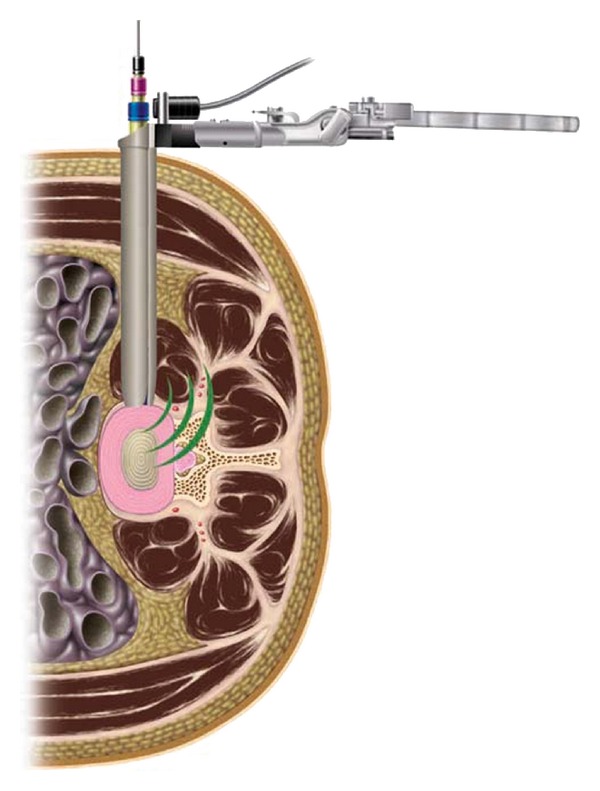
Illustration of XLIF technique.

Recent advances in MIS technology are now being applied to spinal pathologies. One of these techniques, extreme lateral interbody fusion (XLIF) has been suggested as a safe, minimally invasive alternative to traditional open fusion procedures. The technique has previously been described in detail Figure 1 [11] and several reports with long-term outcomes and large-series samples are emerging, showing the efficacy of the approach with fewer morbidities than conventional approaches [12–18].

 XLIF has been recommended for spondylolisthesis up to grade 2 [11, 13] but the concerns about neural complications associated with the lateral approaches to the spine [19–21] beg the question of safety. These concerns are most pronounced at the L4-5 level, where the lumbar plexus is most ventral anatomically [8, 22–27]. Significant anterolisthesis at this level only exacerbates the risk.

 To our knowledge, no reports have specifically addressed the treatment of grade 2 spondylolisthesis at L4-5 with XLIF. Herein, we report on our early and intermediate term results in applying this technique to what is arguably its “worst case scenario.”

## 2. Methods

### 2.1. Patient Population

 Sixty-three patients (10 men and 53 women; mean age 64.5 years) available for 12-month followup after undergoing XLIF for grade 2 spondylolisthesis were treated with XLIF at a single institution between November 2006 and March 2011. In all cases supplemental posterior instrumentation was applied. No posterior direct decompression was performed, relying solely on the indirect decompression achieved through disk height restoration and reduction of slip. Demographics, diagnosis, previous surgery, body mass index (BMI), and preexisting comorbidities were recorded. Under Saint Mary's Health Center Institutional Review Board (IRB) approval, clinical and radiographic outcomes were prospectively collected and evaluated at pre-op, post-op, 3 months, 6 months, and 12 months followup.

### 2.2. Radiographic Evaluation

 Standing anteroposterior (AP), static lateral, and flexion-extension lateral radiographs were obtained preoperatively and at two weeks, three months, six months, and twelve months after surgery. Measurements of disk height (mm) and anterolisthesis (mm) were taken. Spinal stenosis was confirmed by preoperative CT or MR imaging. Radiographic analysis was performed by a physician other than the operating surgeon.

 Fusion was defined as the presence of bridging bone across the disk space (modified Lenke grade 1 or 2) [28] and the absence of significant motion (<5 degrees, <2 mm interspinous widening) on dynamic radiographs. 

### 2.3. Clinical Evaluation

 Visual analog scale (VAS) pain measurements were obtained at each time point through the completion of patient outcomes questionnaires administered by the research staff. Intraoperative and postoperative complications were recorded by the evaluating physician. At twelve months postoperatively, patients were asked to complete an additional questionnaire assessing the presence of new back or leg pain (pain not present prior to surgery), their degree of satisfaction with the result, and their willingness to have the procedure again.

### 2.4. XLIF Surgical Technique

**Figure 2:**
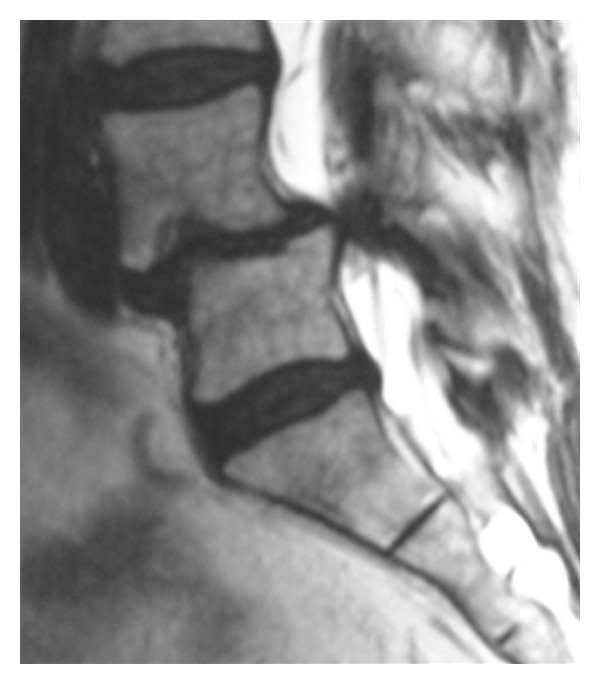
MRI scan showing spinal stenosis and spondylolisthesis.

Extreme lateral interbody fusion, or XLIF, is a 90° off midline or true lateral approach that allows for large graft placement and excellent disk height restoration and provides indirect decompression at the stenotic motion segment. This approach can be performed using two 3 cm to 4 cm skin incisions. Safe passage to the retroperitoneal space is assured by gentle blunt dissection. As the psoas muscle is traversed, the lumbosacral plexus is protected by the use of automated electrophysiology. Exposure is achieved with an expandable three-bladed retractor, which allows for direct illuminated visualization facilitating diskectomy and complete anterior column stabilization using a large load-bearing implant. In patients with significant listhetic deformity, the adherence to procedural technique, including careful patient positioning, gentle retroperitoneal dissection, and meticulous psoas traverse using advanced neurological monitoring before performing a complete discectomy and placing a properly size interbody spacer is essential [13] where neural structures are pulled ventrally by the slipping L4 vertebral body (Figure 2).

**Figure 3:**
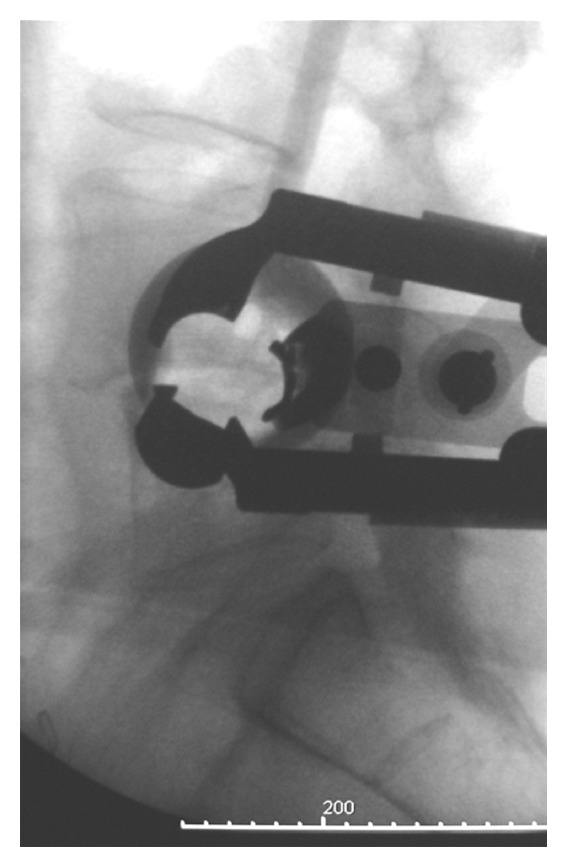
Lateral fluorogram showing dorsal retractor placement.

It is impossible to overemphasize the importance of reliable, timely monitoring of the neural elements as the surgeon traverses the psoas muscle. Visual identification of the lumbar plexus is not possible but the plexus can be protected by using an automated real-time electrophysiology technology (Figure 3).

## 3. Results

The demographic, diagnosis, and comorbidity data for the total cohort are summarized in Table 1. For all patients, hospital stay averaged 1.2 days and hemoglobin decreased 1.4 g on average. There were two (3.4%) complications in the total cohort, one patient experiencing postoperative ileus, the second having a broken pedicle screw on radiographs obtained after a motor vehicle accident 14 months after surgery. CT imaging showed a solid fusion and the patient was asymptomatic. There were no infections. Although early postoperative transient upper thigh pain and hip flexion weakness were common, as expected consequences to operative trauma to the psoas muscle, these symptoms were not persistent. There were no neurologic deficits. Two (3.4%) patients of the total cohort underwent further surgery within one year: both for adjacent segment disease, one treated with PLF, the other with XLIF.

Grade II spondylolistheses were most commonly present at L4-5 (97%), though in a single level each, L2-3, and L3-4 were indicated. A total of 80 levels (1.3 per patient) were treated (63 for grade II spondylolisthesis). One-, two-, and three-level procedures were performed in 78%, 18%, and 5% of cases, respectively. Biologic materials varied, but most included demineralized bone matrix (87%). Transpedicular fixation was used in all but one instance of grade II spondylolisthesis, where transpedicular facet fixation was used. Treatment variables are included in Table 2.

**Figure 4:**
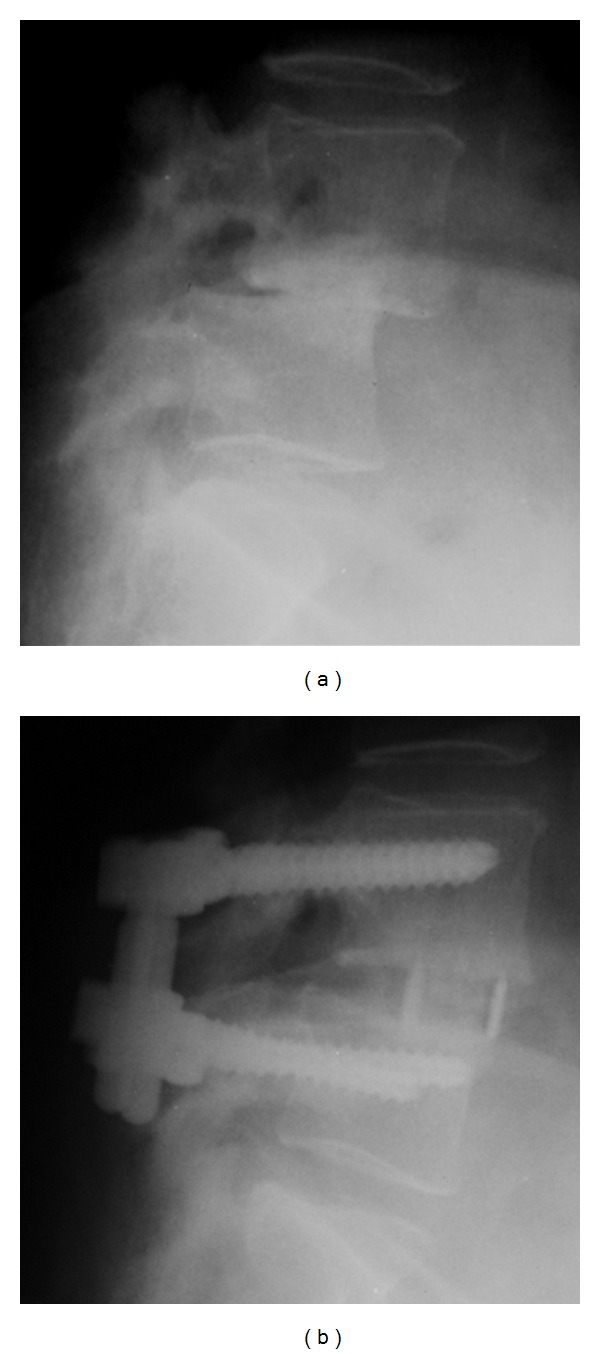
(a) Preoperative lateral radiograph. (b) Lateral radiograph 12-months postoperative.

**Figure 5:**
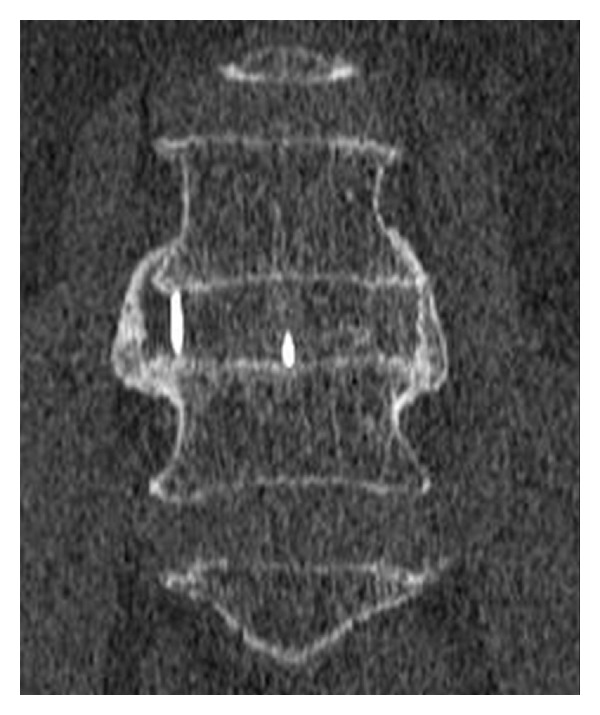
CT image demonstrating fusion.

Clinical and radiographic outcomes are shown in Table 3. At 12 months, there was no radiographic instability noted on dynamic radiographs and all patients appeared to have bridging bone across the interbody space (Figures 4(a) and 4(b)). Eight patients underwent CT imaging. All were judged to be fused by an independent radiologist (Figure 5).

Neither radiographic (slip) nor VAS improvement and maintenance at last followup were influenced by age, BMI/obesity, preexisting comorbidities, prior surgery, levels treated, or unilateral versus bilateral fixation (*P* > 0.05). Patient satisfaction and willingness to have undergone the procedure again were, however, dependent on slip improvement (*P* = 0.011 and *P* = 0.008, resp.). A summary of slip and VAS findings by demographic and treatment variables is included in Table 4. Although average correction was well maintained, 6.4% of patients had lost more than 3 mm of listhetic correction and 6.4% had lost more than 3 mm of disk height.

At last followup, 89.3% rated themselves as “satisfied” or “very satisfied” with their results and 92.9% stated that they would choose to have the procedure again.

## 4. Discussion

 The purpose of this study was to examine the safety and efficacy of XLIF in the treatment of grade 2 spondylolisthesis. The use of XLIF to treat degenerative conditions has been documented, as has the procedure's reduced complication rate when compared to traditional open approaches, either anterior [29] or posterior [30, 31]. Analysis of our results shows excellent reduction in listhetic deformity and improvement of disk height with maintenance of these radiographic outcomes over time. Progression toward fusion appears to be routine. Likewise, clinical outcomes denote marked improvement in VAS with persistent improvement at one year. Patient satisfaction with the procedure approaches 90% in most studies, a finding confirmed by our results. These clinical measures attest to the resolution of stenotic symptoms through the indirect decompression and stabilization achieved.

 However, the concerns regarding the safety of lateral transpsoas approaches to the lumbar spine remain. In a frequently cited study, [19] the authors reported a 27% incidence of groin numbness (but no motor deficits) using an endoscopic transpsoas approach without neurologic monitoring. It should be noted that this study has been mistakenly referenced [19] as a description of the XLIF approach, which is minimally invasive but not endoscopic and mandates the use of real-time neurologic monitoring. Another study [21] noted two L4 nerve root injuries (3.4%) in a series of 58 lateral fusion cases (and a 22.4% complication rate). This paper reported cases using both XLIF and direct lateral interbody fusion (DLIF) without delineating the number of each type of procedure or distinguishing the complications by procedure. Since the recommended technique is somewhat different in the two procedures and the duration of hospitalization was so prolonged (XLIF: 6 days; DLIF: 4 days), one might argue that this study was a learning curve comparison and should not be cited as definitive. In the largest published series to date, 600 patients treated with XLIF experienced a length of hospitalization averaging 1.21 days and a 6.2% complication rate (rate of transient motor deficit—0.7%) [18].

 Nonetheless, neurologic deficits associated with lateral approaches are an area of great discussion. As has been documented anatomically and radiographically, the lumbar plexus migrates ventrally as one descends caudally from L2-3 to L4-5 [8, 22–27]. This places the plexus at greatest risk in a transpsoas approach at the L4-5 level. In addition, anterolisthesis of the superior vertebral body carries the plexus even more ventral, heightening safety concerns. However, as shown by our data, in the presence of real-time neurologic monitoring and with attention to the details of the technique mentioned above, grade 2 listhetic segments, especially at L4-5, can be treated successfully without neurologic injury. The importance of monitoring and technique cannot be overemphasized.

 Clinically, surgery for spondylolisthesis has been shown to yield better patient outcomes than nonoperative treatment in large randomized trials [2–4]. Multiple techniques have been employed—decompression alone, [32] PLF, [6] instrumented PLF [33] PLIF [34], ALIF [35], TLIF [9], as well as MIS ALIF [10], or MIS TLIF [19] procedures—without a clear consensus emerging [5]. In addition to clinical effectiveness, recent results of a randomized controlled trial have shown that instrumented fusion for the treatment of degenerative spondylolisthesis is substantially cost effective compared to conservative care [32]. This study noted a quality-adjusted life-year (QALY) gain of 0.39 in the fusion cohort at a cost of $54,500 (down from 0.23 QALY and $115,600 cost per QALY gained at two years postoperative) per QALY gained. With the threshold for cost effectiveness in the United States at $100,000 per QALY gained, [36, 37] this proves that in the treatment of degenerative spondylolisthesis, instrumented lumbar fusion is solidly cost effective compared to conservative care at four-years postoperative. However, no breakdown of the 344 fusion surgeries (269 with instrumentation) by type of procedure was provided but, based on the timeframe of the study, it may be inferred that the vast majority of those fusions were performed using traditional open techniques. As we have shown in this study, the complications associated with MIS XLIF fusion for spondylolisthesis are notably less than the complications reported with traditional open approaches. Furthermore, open spinal fusions have been reported to have much longer hospitalizations (ALIF: 3.9 days [28], PLIF: 9.7 days [29], or TLIF: 5.5 days [38]) than the 1.2 days we report herein. A recent study, compared the operating costs for a hospital performing XLIF and open PLIF in the treatment of two-level degenerative spinal conditions showed a decrease in operating costs by 9.6% (including the higher price for XLIF implants) with a 1.2 compared with 3.2 day hospital stay (resp.) with significantly fewer transfusions and residual events [39]. A similar study of open and miniopen posterior found significantly lower hospital charges, complications, length of stay, and transfer to inpatient rehabilitation using minimally invasive posterior lumbar interbody fusion (PLIF) compared with open PLIF [40]. It stands to reason that modern surgical fusion options—utilizing direct visualization, miniopen approaches—would be expected to yield a markedly decreased dollar cost per QALY gained because these MIS techniques require shorter hospital stays and result in fewer expensive complications.

## 5. Conclusion

 XLIF is safe and effective for the treatment of grade 2 spondylolisthesis at L4-5. The use of this technique results in marked clinical and radiographic improvement which is maintained over time. The use of real-time neurologic monitoring and careful attention to technique are mandatory.

## Figures and Tables

**Table 1:** Patient demographics of grade II spondylolisthesis patients treated with extreme lateral interbody fusion (XLIF).

Characteristic	Statistic (*n* = 63)
Mean age in years (range)	66.4 (25–88)
Number of females (%)	53 (84.1)
Mean body mass index (BMI) (range)	30.8 (16.9–48.4)
Comorbidities	
Tobacco use (%)	47 (74.6)
Coronary artery disease (%)	39 (61.9)
Diabetes (%)	9 (14.3)
Chronic obstructive pulmonary disease (COPD) (%)	3 (4.8)
Preoperative steroid use (%)	9 (14.3)
Cancer (%)	7 (11.1)
Any prior lumbar surgery (%)	45 (71.4)
Prior surgery type	*n* = 45
Laminectomy (%)	11 (61.1)
Fusion (%)	4 (22.2)
Anterior lumbar interbody fusion (ALIF)	3 (16.7)
Diagnoses (primary only)	
Spondylolisthesis (%)	45 (71.4)
Stenosis with instability (%)	132 (46.6)
Degenerative scoliosis (%)	2 (3.2)

**Table 2:** Treatment characteristics.

Characteristic	Statistic (*n* = 63)
Number of levels treated (average per patient)	80 (1.3)
L2-L3 (% of patients)	2 (3.2)
L3-L4 (% of patients)	15 (23.8)
L4-L5 (% of patients)	61 (96.8)
L5-S1 (AxiaLIF) (% of patients)	2 (3.2)
Number of GII spondy levels (average per patient)	63 (1.0)
Number of total levels treated per case	
One	49 (77.8)
Two	11 (17.5)
Three	3 (4.8)
Graft material	
Beta-TCP/HA (%)	6 (9.5)
DBM + allograft (%)	6 (9.5)
DBM + CCC (%)	49 (77.8)
Allograft cellular bone matrix (%)	2 (3.2)
Supplemental fixation (GII levels)	
Unilateral pedicle screws (%)	53 (84.1)
Bilateral pedicle screws (%)	9 (14.3)
Total pedicle screw fixations (%)	62 (98.4)
Transpedicular facet fixation (%)	1 (1.6)
Internally fixated implant (%)	10 (15.9)
No supplemental fixation (stand alone) (%)	0 (0)
Mean hemoglobin change from pre- to postoperative (g) (range)	−1.4 (−3.8–0.5)
Mean length of hospital stay (days) (range)	1.21 (0–4)

**Table 3:** Average clinical and radiographic outcome data of patients with at least 12 months followup.

	Preop	Postop	3 months	6 months	12 months	*P*
VAS (pain) (stdev.)	8.7 (1.3)		2.2 (2.0)	2.3 (22.0)	2.2 (2.0)	<0.001
Disk height (mm) (stdev.)	4.6 (2.2)	10.3 (2.6)	9.7 (2.6)	9.3 (2.6)	9.0 (2.5)	<0.001
Slip (mm) (stdev.)	11.1 (1.7)	3.0 (2.0)	3.3 (2.2)	3.6 (2.3)	3.6 (2.3)	<0.001
Lenke			1.9 (0.5)	1.4 (0.4)	1.1 (0.3)	

Mm: millimeters, stdev: standard deviation.

**Table 4:** Breakdown of spondylolisthesis reduction and 12-month pain (VAS) by demographic and treatment variables.

Grouping variable (*n*)	Slip reduction from preoperative (%)			12-month VAS (mm)		
	No	Yes	*P*	No	Yes	*P*
Gender (female/male)	67% (53)	75% (10)	0.228	2.2 (48)	2.1 (10)	0.933
Obese	68% (36)	68% (27)	0.925	2.1 (24)	2.2 (34)	0.840
Smoke	68% (47)	67% (16)	0.862	2.1 (44)	2.2 (14)	0.915
Diabetes mellitus	69% (54)	62% (9)	0.345	2.1 (50)	2.4 (8)	0.677
Coronary artery disease	68% (39)	68% (24)	0.934	1.9 (34)	2.5 (24)	0.249
Chronic obstructive pulmonary disease	69% (60)	56% (3)	0.273	2.2 (55)	2.3 (3)	0.856
Steroid use	67% (54)	76% (9)	0.149	2.1 (49)	2.2 (9)	0.916
Cancer	68% (56)	70% (7)	0.714	2.2 (52)	2.0 (6)	0.760
Prior surgery	69% (45)	66% (18)	0.680	2.1 (43)	2.3 (15)	0.800
Levels treated (1 or 2)	67% (49)	71% (11)	0.637	2.1 (44)	2.6 (11)	0.508
Unilateral versus bilateral fixation (uni/bi)	68% (53)	70% (8)	0.799	2.3 (49)	1.7 (7)	0.445
Satisfaction	49% (6)	70% (50)	**0.011**	5.7 (6)	1.7 (45)	**0.016**
Redo	43% (4)	70% (52)	**0.008**	5.3 (4)	1.8 (47)	**0.041**

VAS: visual analog scale, mm: millimeters, uni: unilateral, and bi: bilateral.

## References

[1] Kalanithi PS, Patil CG, Boakye M (2009). National complication rates and disposition after posterior lumbar fusion for acquired spondylolisthesis. *Spine*.

[2] Pearson AM, Lurie JD, Blood EA (2008). Spine patient outcomes research trial: radiographic predictors of clinical outcomes after operative or nonoperative treatment of degenerative spondylolisthesis. *Spine*.

[3] Weinstein JN, Lurie JD, Tosteson TD (2007). Surgical versus nonsurgical treatment for lumbar degenerative spondylolisthesis. *The New England Journal of Medicine*.

[4] Weinstein JN, Lurie JD, Tosteson TD (2008). Surgical versus nonoperative treatment for lumbar disc herniation: four-year results for the Spine Patient Outcomes Research Trial (SPORT). *Spine*.

[5] Resnick DK, Choudhri TF, Dailey AT (2005). Guidelines for the performance of fusion procedures for degenerative disease of the lumbar spine. Part 9: fusion in patients with stenosis and spondylolisthesis. *Journal of Neurosurgery Spine*.

[6] Sengupta DK, Herkowitz HN (2005). Degenerative spondylolisthesis: review of current trends and controversies. *Spine*.

[7] Tosteson AN, Tosteson TD, Lurie JD Factors Affecting 4-year cost-effectiveness of surgery for stenosis with or without degenerative spondylolisthesis in the Spine Patient Outcomes Research Trial (Sport).

[8] Moro T, Kikuchi SI, Konno SI, Yaginuma H (2003). An anatomic study of the lumbar plexus with respect to retroperitoneal endoscopic surgery. *Spine*.

[9] Lauber S, Schulte TL, Liljenqvist U, Halm H, Hackenberg L (2006). Clinical and radiologic 2 - 4-Year results of transforaminal lumbar interbody fusion in degenerative and isthmic spondylolisthesis grades 1 and 2. *Spine*.

[10] Kim JS, Kang BU, Lee SH (2009). Mini-transforaminal lumbar interbody fusion versus anterior lumbar interbody fusion augmented by percutaneous pedicle screw fixation: a comparison of surgical outcomes in adult low-grade isthmic spondylolisthesis. *Journal of Spinal Disorders and Techniques*.

[11] Ozgur BM, Aryan HE, Pimenta L, Taylor WR (2006). Extreme Lateral Interbody Fusion (XLIF): a novel surgical technique for anterior lumbar interbody fusion. *Spine Journal*.

[12] Ozgur BM, Agarwal V, Nail E, Pimenta L (2010). Two-year clinical and radiographic success of minimally invasive lateral transpsoas approach for the treatment of degenerative lumbar conditions. *SAS Journal*.

[13] Rodgers WB, Cox CS, Gerber EJ (2007). Experience and early results with a minimally invasive technique for anterior column support through extreme lateral interbody fusion (XLIF). *US Musculoskeletal Review*.

[14] Rodgers WB, Cox CS, Gerber EJ (2009). Minimally invasive treatment (XLIF) of adjacent segment disease after prior lumbar fusions. *The Internet Journal of Minimally Invasive Spinal Technology*.

[15] Rodgers WB, Cox CS, Gerber EJ (2010). Early complications of extreme lateral interbody fusion in the obese. *Journal of Spinal Disorders and Techniques*.

[16] Rodgers WB, Gerber EJ, Patterson JR (2010). Fusion after minimally disruptive anterior lumbar interbody fusion: analysis of extreme lateral interbody fusion by computed tomography. *SAS Journal*.

[17] Rodgers WB, Gerber EJ, Rodgers JA (2010). Lumbar fusion in octogenarians: the promise of minimally invasive surgery. *Spine*.

[18] Rodgers WB, Gerber EJ, Patterson J (2011). Intraoperative and early postoperative complications in extreme lateral interbody fusion: an analysis of 600 cases. *Spine*.

[19] Bergey DL, Villavicencio AT, Goldstein T, Regan JJ (2004). Endoscopic lateral transpsoas approach to the lumbar spine. *Spine*.

[20] Booth KC, Bridwell KH, Eisenberg BA, Baldus CR, Lenke LG (1999). Minimum 5-year results of degenerative spondylolisthesis treated with decompression and instrumented posterior fusion. *Spine*.

[21] Knight RQ, Schwaegler P, Hanscom D, Roh J (2009). Direct lateral lumbar interbody fusion for degenerative conditions: early complication profile. *Journal of Spinal Disorders and Techniques*.

[22] Benglis DDM, Vanni S, Levi AD (2009). An anatomical study of the lumbosacral plexus as related to the minimally invasive transpsoas approach to the lumbar spine: laboratory investigation. *Journal of Neurosurgery Spine*.

[23] Hu WK, He SS, Zhang SC (2011). An MRI study of psoas major and abdominal large vessels with respect to the X/DLIF approach. *European Spine Journal*.

[24] Kepler CK, Bogner EA, Herzog RJ, Huang RC (2011). Anatomy of the psoas muscle and lumbar plexus with respect to the surgical approach for lateral transpsoas interbody fusion. *European Spine Journal*.

[25] Park DK, Lee MJ, Lin EL, Singh K, An HS, Phillips FM (2010). The relationship of intrapsoas nerves during a transpsoas approach to the lumbar spine: anatomic study. *Journal of Spinal Disorders and Techniques*.

[26] Regev GJ, Chen L, Dhawan M, Lee YP, Garfin SR, Kim CW (2009). Morphometric analysis of the ventral nerve roots and retroperitoneal vessels with respect to the minimally invasive lateral approach in normal and deformed spines. *Spine*.

[27] Uribe JS, Arredondo N, Dakwar E, Vale FL (2010). Defining the safe working zones using the minimally invasive lateral retroperitoneal transpsoas approach: an anatomical study. *Journal of Neurosurgery Spine*.

[28] Lenke LG, Bridwell KH, Bullis D, Betz RR, Baldus C, Schoenecker PL (1992). Results of in situ fusion for isthmic spondylolisthesis. *Journal of Spinal Disorders*.

[29] Sasso RC, Best NM, Mummaneni PV, Reilly TM, Hussain SM (2005). Analysis of operative complications in a series of 471 anterior lumbar interbody fusion procedures. *Spine*.

[30] Carreon LY, Puno RM, Dimar JR, Glassman SD, Johnson JR (2003). Perioperative complications of posterior lumbar decompression and arthrodesis in older adults. *Journal of Bone and Joint Surgery A*.

[31] DiPaola CP, Molinari RW (2008). Posterior lumbar interbody fusion. *Journal of the American Academy of Orthopaedic Surgeons*.

[32] Tosteson ANA, Lurie JD, Tosteson TD (2008). Surgical treatment of spinal stenosis with and without degenerative spondylolisthesis: cost-effectiveness after 2 years. *Annals of Internal Medicine*.

[33] Kimura I, Shingu H, Murata M, Hashiguchi H (2001). Lumbar posterolateral fusion alone or with transpedicular instrumentation in L4-L5 degenerative spondylolisthesis. *Journal of Spinal Disorders*.

[34] Suk SI, Lee CK, Kim WJ, Lee JH, Cho KJ, Kim HG (1997). Addding posterior lumbar interbody fusion to pedicle screw fixation and posterolateral fusion after decompression in spondylolytic spondylolisthesis. *Spine*.

[35] Min JH, Jang JS, Lee SH (2007). Comparison of anterior- and posterior-approach instrumented lumbar interbody fusion for spondylolisthesis. *Journal of Neurosurgery Spine*.

[36] Laupacis A, Feeny D, Detsky AS, Tugwell PX (1992). How attractive does a new technology have to be to warrant adoption and utilization? Tentative guidelines for using clinical and economic evaluations. *CMAJ*.

[37] Laupacis A, Feeny D, Detsky AS, Tugwell PX (1993). Tentative guidelines for using clinical and economic evaluations revisited. *CMAJ*.

[38] Dhall SS, Wang MY, Mummaneni PV (2008). Clinical and radiographic comparison of mini-open transforaminal lumbar interbody fusion with open transforaminal lumbar interbody fusion in 42 patients with long-term follow-up: Clinical article. *Journal of Neurosurgery Spine*.

[39] Deluzio KJ, Lucio JC, Rodgers WB (2010). Value and cost in less invasive spinal fusion surgery: lessons from a community hospital. *SAS Journal*.

[40] Wang MY, Cummock MD, Yu Y, Trivedi RA (2010). An analysis of the differences in the acute hospitalization charges following minimally invasive versus open posterior lumbar interbody fusion. *Journal of Neurosurgery Spine*.

